# Factors Associated with Cultivation of Tobacco in Bangladesh: A Multilevel Modelling Approach

**DOI:** 10.3390/ijerph17124277

**Published:** 2020-06-15

**Authors:** Ashis Talukder, Iqramul Haq, Mohammad Ali, Jeffrey Drope

**Affiliations:** 1 Statistics Discipline, Khulna University, Khulna 9208, Bangladesh; ali@ku.ac.bd; 2 Dept. of Agricultural Statistics, Sher-e-Bangla Agricultural University, Dhaka 1207, Bangladesh; iqramul.haq@sau.edu.bd; 3 Scientific Vice President, Economic and Health Policy Research, American Cancer Society, 250 Williams Street, Atlanta, GA 30303, USA; jeffrey.drope@cancer.org

**Keywords:** tobacco farming, economic livelihoods, Bangladesh

## Abstract

An increasing number of studies provide evidence on the serious negative consequences of tobacco farming on economic livelihoods, human health and the environment. There is, however, only limited research on tobacco farming in Bangladesh, a significant producer of tobacco leaf. It is not yet well understood why many farmers choose to grow tobacco considering the challenging context. Accordingly, this study examines the factors that influence farmers’ decisions to grow tobacco in Bangladesh. Socio-demographic and economic information was collected from 220 tobacco farmers and 117 non-tobacco farmers from the major tobacco-growing district of Kushtia, for a total sample of 337. These farmers were recruited from two sub-districts (or *upazilla*—Daulatpur and Mirpur) using a stratified random sampling. A two-level logistic regression model was applied for the identification of the variables that condition farmers’ decisions to cultivate tobacco leaf. Almost two-thirds of the sampled farmers (65.3%) chose to farm tobacco. The results demonstrate that the following variables shape most farmers’ decisions to cultivate tobacco: older age, less education, tobacco firms’ short-term financial support of growing tobacco, greater ease of selling tobacco products at market, better access to credit (also provided by the tobacco companies), and farmer’s perception about higher profits from tobacco cultivation compared to other crops. This study strongly suggests that the government and others working on tobacco control should consider engaging in initiatives to increase farmers’ education, perhaps particularly for older farmers, and provide meaningful financial support in part by helping to increase access to credit and ensuring a better market facility to sell their other healthier agricultural crops, goods and services.

## 1. Introduction

Tobacco use is a major risk factor for developing the most common non-communicable diseases and continues to be a major global public health concern. Worldwide, more than eight million deaths occur from tobacco use annually [1]. The burden of tobacco use in Bangladesh is staggering, killing at least 126,000 annually with the total economic cost each year estimated at BDT 305.6 billion ($3.61 billion), equivalent to 1.4% of the GDP of Bangladesh in 2017–2018 [2]. Research demonstrates that more than one third (35%) of Bangladeshi adults use tobacco products, and/or combustible smokeless products [3]. What is also alarming is the fact that 39% and 43% adults are exposed to secondhand smoke in their homes and workplaces, respectively [3]. This secondhand smoke contains hundreds of toxic chemicals, of which at least 70 are carcinogenic [4,5,6,7]. 

Despite the known harms and the commitment by many health ministries to strengthen tobacco control, tobacco leaf remains an important agricultural commodity in many countries, including in Bangladesh. The economics of tobacco production create barriers to strengthen tobacco control measures because elected officials slow or stop public health efforts ostensibly because of a fear of hurting these farmers. In Bangladesh, tobacco cultivation has a long history. Large-scale tobacco farming has occurred since the 1960s, even when rice and other food crops were the main agricultural products [8]. After liberation in 1971, tobacco farming spread widely in Teesta silt in the Rangpur region, supported by the British American Tobacco Company [8]. Several studies were conducted by the Bangladesh Agricultural Research Institute (BARI) to understand the development activities of tobacco and they finally recommended to abandon cultivation in 1995 [8]. However, tobacco cultivation continues largely through contract growing with multinational tobacco companies, including with the British American Tobacco Company [8].

There is a growing body of literature on tobacco farming particularly in tobacco-growing low- and middle-income countries, including in Bangladesh. Some studies provide evidence of the serious consequences of tobacco farming on human health and the environment [9,10,11,12,13]. For example, the basic agricultural processes associated with tobacco farming lead to deforestation and soil degradation [9]. Moreover, agrochemical contamination leads to environmental interruptions that cause a negative impact on the ecosystem, including biodiversity, land resources, and food sources. More germane to this research, recent studies in different tobacco-growing countries have examined the political economy of tobacco production [14,15], the economic livelihoods of tobacco farmers [16,17,18,19,20], the factors that shape farmers’ decisions to grow tobacco, and economic alternatives to tobacco production. In Bangladesh, a recent study examining the factors associated with tobacco farming found that the variables most shaping farmers’ decisions to continue growing tobacco were: tobacco firms’ incentives to farmers, farmers’ perceived profitability of tobacco growing, a “guaranteed” market for tobacco crops, and a perceived overall economic viability [16]. We build on this research by examining the factors associated with tobacco farming in a different Bangladeshi regions using a novel sample of both tobacco farmers and their neighbors who were not growing tobacco, with a corresponding appropriate methodology of two-level regression which is important for controlling for possible regional effects that exist in tobacco leaf production.

## 2. Materials and Methods

### 2.1. Data Collection

Primary data were collected using a sample survey. The research team developed a questionnaire based on an intensive literature review [11,12,17,18]. The questionnaire was developed to collect relevant information including: farmer type (tobacco farmer or non-tobacco farmer), respondent’s age, education level, farming experience, size of cultivated land, perception of profit status, short term benefit of tobacco cultivation, market facility, and better access to credit provided by tobacco companies. To collect samples, at first we selected two major tobacco-producing upazilla (upzilla is an administrative region in Bangladesh that functions as a sub-unit of a district), Daulatpur, and Mirpur, purposively based on more intensive tobacco growing. These two upazilla have 37,246 hectares (91%) of their land under tobacco cultivation in the Kushtia district [19]. After identifying the area, we contacted the corresponding district agricultural office (Agricultural offices collect information related to agriculture such as production rate, number of farmers in the district etc., and promote policies to improve the current conditions of farmers) in each upazilla and collected a comprehensive list of farmers. After the collection of the farmers’ lists, we considered each upazilla as a stratum. Then, a random number generator was considered and selected 355 farmers as a sample. After selecting the farmers, two of the authors went to the farmers’ houses and collected data in face-to-face interviews with farmers. Ultimately, a total of 337 farmers who fully completed the questionnaires (response rate of 94.9%) were included in the final analysis. Among them, 220 farmed tobacco and 117 did not. Note that the distribution of surveyed farmers among the two upazilla, Daulatpur and Mirpur are 57% and 43%, respectively. 

### 2.2. Variable Selection

The adoption status of tobacco cultivation (1, if the farmer adopted tobacco cultivation, 0, otherwise) is the dependent variable. The farmer is considered a tobacco grower if he/she adopted tobacco cultivation in the previous year; otherwise, the farmer is considered to be a non-tobacco grower. Drawing from the literature on farmers’ decisions to grow, the independent variables are: respondents’ age (<30 years, 30–34 years, 35–40 years, >40 years), education level (primary or less education, secondary, or higher education), farming experience (less than 10 years of farming experience, more than 10 years), the farmer’s perception of profit status of tobacco cultivation (yes, if the farmer thinks that tobacco cultivation is profitable; no, if the farmer does not think so), farm size (less than 1 acre, 1–1.5 acre, more than 1.5 acre), the farmer’s perception of short-term financial benefit of tobacco farming (yes, no), better access to credit provided by tobacco companies (yes, no), market facility provided by tobacco companies (yes, no), and the upazilla (Daulatpur and Mirpur).

### 2.3. Statistical Analysis

To examine the determinants of tobacco cultivation, we applied a two-level logistic regression model to reduce any regional effect (the effect due to the different upazilla in the Kushtia district). This is the first research on farming in Bangladesh to apply this methodology. The mathematical form of the two level logistic regression model can be defined as follows:

$$logit\;\left(\pi_{ij}\right)=\alpha_{00}+\alpha_{10}x_{ij}+\alpha_{01}X_{j}+U_{oj}$$

where $logit\;\left(\pi_{ij}\right)=P\left(Y_{IJ}=1\right),\;\alpha_{00}$ is the fixed effect intercept and $U_{0j}$ is the level-2 residual. Moreover, $x_{ij}$ and $X_{J}$ refers to level-1 and level-2, respectively, whereas $\alpha_{10}$ and $\alpha_{01}$ refers to the fixed effects of level-1 and level-2 variable respectively. Note that, in this research, all the selected covariates are classified as individual level variables (Level-1) or regional level variables (Level-2). Regional level variables consist of upazillas of Kushtia district (Daulatpur and Mirpur) and individual level variables include the remaining covariates. 

Before applying any multilevel model, intra-class correlation coefficient (ICC) should be calculated. A multilevel logistic regression model can be applied if the ICC is greater than 0 [20]. In this study, the multilevel empty model is considered to calculate ICC, and a two-level random intercept model is applied to identify the possible risk factors for tobacco cultivation. 

### 2.4. Ethical Statement

All participants of this study were informed prior to data collection about the purpose of the study. Verbal consent was taken from the farmers prior to data collection. Participant anonymity and confidentiality of data were ensured, and then participants were provided with information about the nature and purpose of the study, the procedure, and the right to withdraw their data from the study. The study protocol was taken from the academic review committee of Statistics Discipline, Khulna University, Bangladesh. The reference number is: R-005 (1)/2018; Date: 15 May 2018.

## 3. Results 

### 3.1. Demographic Characteristics 

The average age of the sampled farmers is 38 years with a standard deviation (SD) of six years. It is observed that among 337 individuals surveyed, 47.3% had primary or less education, and 52.7% of respondents had some secondary or higher education. More than half of the farmers (54%) had more than 10 years of farming experience. Most of the farmers (53%) had 1 to 1.5 acres of cultivated land, while 20% had less than 1 acre and 27% had more than 1.5 acres of cultivated land. Finally, almost two-thirds of the sampled farmers (65.3%) reported cultivating tobacco.

### 3.2. Factors Associated with the Decision to Farm Tobacco 

To identify the factors associated with tobacco cultivation, we have applied a two-level logistic regression model. Before fitting this model, we calculated the value of the ICC within upazillas by fitting a two-level empty model. The results are shown in Table 1. From this table, it is identified that the value of the ICC is 0.26, indicating that there exists about 26% heterogeneity between the two upazillas and a two-level regression model can be applied in this study. Accordingly, we have applied a two-level random intercept model for identifying the factors associated with tobacco cultivation in Bangladesh. The maximum likelihood estimates and odds ratios of this model are displayed in Table 2. From this table, we observe that the age of the farmer has a positive effect on tobacco cultivation. That is, as the age of the farmer increases, the odds of growing tobacco also increase in a significant way (p<0.01). To be specific, the odds of choosing to cultivate tobacco are 8.95 times higher (OR = 8.953, p<0.01) for the farmers belonging to the age group (35–39), compared to farmers younger than 30 years old. The odds are 9.72 times higher for the farmers aged 40 years and older. The results demonstrate that farmers with more education are less likely to cultivate tobacco than those with no or primary education. The odds ratios also suggest that the short-term financial benefit of tobacco farming, better market facility, and better access to credit provided by tobacco companies have positive and statistically significant effects on tobacco farming. The farmers who thought tobacco cultivation was profitable have a greater likelihood of choosing tobacco farming. Moreover, among the regions, the odds of electing to farm tobacco is higher in Daulatpur upazilla. Finally, farming experience and farm size have no significant effect on the decision to cultivate tobacco in this analysis (p>0.10).

## 4. Discussion

Based on the regression model, we found that more educated farmers have a lower probability of tobacco farming. The results also suggest that the short-term financial benefit of tobacco cultivation has a significant impact on whether farmers decide to grow tobacco in Bangladesh, similar to other studies [15,16,20,21,22]. This dynamic is at least two-fold: tobacco farming can provide opportunities to generate cash, which households need for certain goods and services, and there is a perception that this cash can be generated in a relatively short time frame from farming tobacco compared to farming other crops. Better market facility and better access to credit provided by tobacco companies also appear to have positive effects on decisions to grow tobacco in this study, also consistent with previous studies conducted in Bangladesh [8,10,23]. In terms of market dynamics, several tobacco companies guarantee the purchase of the tobacco crops produced by farmers. Accordingly, the farmers feel secure that if they choose to grow tobacco leaf, they will at least receive payment for their product, even if they cannot be sure how high the price will be. In addition, farmers’ perceptions of higher profits from selling tobacco attract some farmers to tobacco, which is a finding that is similar to previous studies in Bangladesh [8]. The finding that older farmers are more likely to grow tobacco is consistent with other research around the world, including in other Asian countries, and among other variables it is often related to preferring to continue with a crop they believe they know how to grow well and because they believe it is one of the few ways to generate cash [24,25]. Qualitative interviews with Bangladeshi farmers could enhance the understanding of these findings, and is an important avenue for future research. Farmers in Daulatpur upazilla are more likely to farm tobacco leaf, but it is not clear which variables are shaping this dynamic, requiring further investigation of the characteristics of the sub-district, which is beyond the purview of this study. 

This study has several limitations. First, there are potentially other factors (for example, price stability, sales-production ratios, sales guarantee of tobacco, etc.) not included in this analysis that influence farmers’ decisions to cultivate tobacco. Because of limited money and time, we could not include every conceivable factor in our research. That said, we drew from the existing literature to include as many variables as possible that other research has found to be pertinent. Second, we have a non-response rate of about 5% that reduced our sample size. Third, the study only examined one major district in the country. We are reassured that the findings were congruent with previous research in other parts of the country, so collectively, these studies are furnishing a clear illustration of this important dynamic in Bangladesh.

## 5. Conclusions

The burden of tobacco use in Bangladesh continues to be enormous. To reduce this burden, tobacco farming should be minimized by shifting farmers’ motivations for growing tobacco. This study identifies several important variables associated with farmers choosing to grow tobacco in Bangladesh, which is information that is vital to understanding how government and other actors can help farmers make this transition. For example, programs that ensure better market facility to sell non-tobacco crops, which are not harmful to health or to the environment, would be a logical first step. Similarly, programs that enhance smallholder farmers’ access to credit would be another obvious partial but potentially fruitful solution. While the age of farmers obviously cannot be changed, perhaps education programs to introduce longtime farmers to alternative crops is a viable program. Regardless of what type of program governments choose to undertake, all efforts must include farmers, for example, through their associations, to make certain that the initiative matches their needs and are eminently executable. These efforts cannot realistically include the tobacco industry, which has a strong vested interest in a status quo that exploits farmers. Because of the policy complexity of these tasks, it is likely going to require a joint effort of the Ministry of Health, the Ministry of Agriculture, the Ministry of Finance, and the Ministry of Environment to reduce tobacco farming in Bangladesh. Finally, the lessons learned in this research are remarkably similar to the experiences of a handful of other countries cited here (e.g., Indonesia, Kenya, Malawi, Philippines, and Zambia). A consistent and increasingly robust body of evidence is rapidly emerging that elucidates both what is undermining smallholder tobacco farmers economically, and what stakeholders, including especially government, can do to mitigate these issues and improve livelihoods.

## Figures and Tables

**Table 1:** Maximum likelihood estimates of multilevel empty model with intra class correlation coefficient (ICC).

Covariates	Estimate	Odds Ratio	*p*-Value
**Fixed effect**			
Intercept	0.658	1.930	0.422
**Random effect**			
$\delta^{2}{}_{u0}$ (Intercept)	1.144	-	<0.03
**Intra class correlation coefficient (ICC)**			
ICC = $\frac{1.144}{\left(1.144\;+\;3.289\right)}=0.26$			

**Table 2:** Maximum likelihood estimates of multilevel random intercept logistic regression model.

Covariates	Estimate	Odds Ratio (95% CI)	*p*-Value
**Fixed effects**			
Intercept	−3.987	0.018 (0.015, 0.147)	<0.001
**Age Group (in months)(reference: <30 years)**			
30–34	0.808	2.243 (0.576, 4.741)	0.243
35–39	2.192	8.953 (2.021, 12.032)	<0.01
40+	2.275	9.727 (2.378, 11.203)	<0.01
**Education Level (reference: No Education)**			
Secondary or higher	−2.389	0.091 (0.032, 0.260)	<0.001
**Farming Experience (referecnce: At least 10 years)**			
More than 10 years	0.019	1.019 (0.335, 3.099)	0.973
**Farm Size (reference: <1 acre)**			
1–1.5 acre	−0.911	0.402 (0.111, 1.453)	0.164
>1.5 acre	−0.922	0.397 (0.133, 1.187)	0.098
**Farmer’s Perception about Profit Status of Tobacco Cultivation (reference: No)**			
Yes	2.061	7.853 (1.906, 13.390)	<0.01
**Short-Term Financial Benefit (reference: No)**			
Yes	1.544	4.683 (1.861, 11.786)	<0.01
**Market Facility Provided by Tobacco Companies (reference: No)**			
Yes	1.167	3.212 (1.360, 7.594)	<0.01
**Better Access to Credit Provided by Tobacco Companies (reference: No)**			
Yes	1.812	6.122 (4.231, 14.223)	<0.001
**Upizilla (reference: Mirpur)**			
Daulatpur	1.988	7.301 (2.883, 15.356)	<0.001

## References

[1] (2019). World Health Organization. WHO Report on Tobacco Control in Bangladesh, Country Office for Bangladesh.

[2] Faruque G.M., Ahmed M., Huq I., Parven R., Wadood S.N., Chowdhury S.R., Hussain A.K.M.G., Haifley G., Drope J., Nargis N. (2020). The Economic Cost of Tobacco Use in Bangladesh: A Health Cost Approach.

[3] World Health Organization (2009). Global Adult Tobacco Survey: Bangladesh Report. Country Office for Bangladesh. https://www.who.int/tobacco/surveillance/global_adult_tobacco_survey_bangladesh_report_2009.pdf?ua.

[4] U.S. Department of Health and Human Services The Health Consequences of Smoking—50 Years of Progress: A Report of the Surgeon General. https://www.ncbi.nlm.nih.gov/pubmed/24455788.

[5] U.S. Department of Health and Human Services The Health Consequences of Involuntary Exposure to Tobacco Smoke: A Report of the Surgeon General. https://www.ncbi.nlm.nih.gov/books/NBK44324/.

[6] U.S. Department of Health and Human Services A Report of the Surgeon General: How Tobacco Smoke Causes Disease: What It Means to You. https://www.cdc.gov/tobacco/data_statistics/sgr/2010/consumer_booklet/pdfs/consumer.pdf.

[7] U.S. Department of Health and Human Services Let’s Make the Next Generation Tobacco-Free: Your Guide to the 50th Anniversary Surgeon General’s Report on Smoking and Healthpdf Iconexternal Icon. https://www.hhs.gov/sites/default/files/consequences-smoking-consumer-guide.pdf.

[8] Hossain M.M., Rahman M.M. (2013). A socioeconomic analysis on tobacco cultivation in Kushtia district of Bangladesh. Soc. Sci..

[9] Lecours N., Almeida G.E.G., Abdallah J.M., Novotny T.E. (2012). Environmental health impacts of tobacco farming: A review of the literature. Tob. Control.

[10] Naher F., Efroymson D. (2007). Tobacco Cultivation and Poverty in Bangladesh: Issues and Potential Future Directions.

[11] Choudhury K., Hanifi S.M.A., Mahmood S.S., Bhuiya A. (2007). Sociodemographic characteristics of tobacco consumers in a rural area of Bangladesh. J. Health Popul. Nutr..

[12] Ali Y., Islam M.F., Rahman M.R., Sheema M.K., Akhtar M.R. (2015). Tobacco farming in Bangladesh and its impact on environment. IOSR J. Environ. Sci. Toxicol. Food Technol..

[13] Efroymson D., Pham H.A., Jones L., Fitz Gerald S., Thu le T., Hien le T.T. (2011). Tobacco and poverty: Evidence from Vietnam. Tob. Control.

[14] Fang J., De Souza L., Smith J., Lee K. (2020). “All Weather Friends”: How China Transformed Zimbabwe’s Tobacco Sector. Int. J. Environ. Res. Public Health.

[15] Makoka D., Drope J., Appau A., Labonte R., Li Q., Goma F., Lencucha R. (2017). Costs, revenues and profits: An economic analysis of smallholder tobacco farmer livelihoods in Malawi. Tob. Control.

[16] Rahman M.S., Ahmed N.F., Ali M., Abedin M.M., Islam M.S. (2019). Determinants of tobacco cultivation in Bangladesh. Tob. Control.

[17] Hasan K.M. (2019). Factors influencing farmers turning into tobacco cultivation in the Khulna division of Bangladesh: An empirical study. Tob. Prev. Cessat..

[18] Rahman M.Z. (2017). Determinants of Extent of Tobacco Cultivation Area in Kustia District.

[19] Policy Research for Development Alternative (2010). Farmers against Tobacco Cultivation: Say ‘No’ to Tobacco Cultivation.

[20] Appau A., Drope J., Witoelar F., Chavez J.J., Lencucha R. (2019). Why do farmers grow tobacco? A qualitative exploration of farmer’s perspectives in Indonesia and Philippines. Int. J. Environ. Res. Public Health.

[21] Appau A., Drope J., Goma F., Magati P., Labonte R., Makoka D., Lencucha R. (2019). Explaining why farmers grow tobacco: Evidence from Malawi, Kenya, and Zambia. Nicotine Tob. Res..

[22] Park S., Lake E.T. (2005). Multilevel modeling of a clustered continuous outcome: Nurses’ work hours and burnout. Nurs. Res..

[23] Magati P., Lencucha R., Li Q., Drope J., Labonte R., Appau A.B., Makoka D., Goma F., Zulu R. (2019). Costs, contracts and the narrative of prosperity: An economic analysis of smallholder tobacco farming livelihoods in Kenya. Tob. Control.

[24] Drope J., Li Q., Araujo E.C., Harimurti P., Sahadewo G.A., Nargis N., Durazo J., Witoelar F., Sikoki B.S. (2018). The Economics of Tobacco Farming in Indonesia.

[25] Chavez J.J., Drope J., Li Q., Aloria M.J. (2016). The Economics of Tobacco Farming in the Philippines. Quezon City, Action for Economic Reforms and Atlanta: American Cancer Society. http://aer.ph/industrialpolicy/wp-content/uploads/2016/09/REPORT-The-Economics-of-Tobacco-Farming-in-the-Philippines-LAYOUT.Pdf.

